# Reliability of differential time to positivity technique for diagnosing catheter-related bloodstream infections: a retrospective analysis

**DOI:** 10.1128/spectrum.02678-24

**Published:** 2025-04-08

**Authors:** Álvaro Irigoyen-von-Sierakowski, Marta Díaz-Navarro, Andrés Visedo, María Jesús Pérez-Granda, Pablo Martín-Rabadán, Patricia Muñoz, María Guembe

**Affiliations:** 1 Department of Clinical Microbiology and Infectious Diseases, Hospital General Universitario Gregorio Marañón 16483, Madrid, Spain; 2 Instituto de Investigación Sanitaria Gregorio Marañón 559924 , Madrid, Spain; 3 CIBER Enfermedades Respiratorias-CIBERES (CB06/06/0058), Madrid, Spain; 4 School of Medicine, Universidad Complutense de Madrid, Madrid, Spain; NHLS Tygerberg/Stellenbosch University, Cape Town, Western Cape, South Africa

**Keywords:** bacteremia, catheter, differential time to positivity, catheter culture, outcome

## Abstract

The differential time to positivity (DTTP) technique is the recommended conservative procedure to diagnose catheter-related bloodstream infection (C-RBSI). However, its reliability and accuracy remain under debate. Therefore, we aimed to compare the DTTP technique feasibility to detect C-RBSI compared to the catheter culture (CC) method. We conducted a 9-month retrospective study including bacteremic episodes in which both DTTP blood cultures (BC) and CC were obtained. We analyzed the diagnostic validity of the DTTP technique for detecting C-RBSI compared to the gold standard (C-RBSI with CC), along with patient clinical data. We included 37 episodes of C-RBSI where both DTTP BC and CC were obtained. C-RBSI was confirmed by both techniques in only 13 episodes (35.1%), whereas in 11 (29.7%) and 13 (35.1%), only DTTP BC or DTTP BC with CC (with a difference between catheter lumen and peripheral BC growth of <2 hours) was positive, respectively. Therefore, the validity values of the DTTP technique for predicting C-RBSI were as follows: sensitivity, 50.0%; specificity, 71.8%; positive predictive value, 54.2%; and negative predictive value, 68.3%. The distribution of microorganisms was similar among the three groups. All patients in whom colonization was not demonstrated by CC (*n* = 11) had been receiving antibiotics before catheter withdrawal. DTTP is a conservative technique that might help to diagnose C-RBSI mostly in situations where catheter removal cannot be achieved. However, it should be interpreted with caution and never be used to rule out C-RBSI. CC before starting antimicrobial therapy remains the most reliable method to diagnose and confirm an episode of C-RBSI.

**IMPORTANCE:**

We try to clarify the reliability of the differential time to positivity technique to predict C-RBSI. It may be interpreted with caution and considering clinical signs, as some C-RBSI can be misdiagnosed.

## INTRODUCTION

Intravenous devices are currently a fundamental tool for the delivery of fluids, drugs, blood products, and parenteral nutrition and for monitoring hemodynamic status and providing hemodialysis. One major complication associated with the use of these devices is local or systemic infections, particularly catheter-related bloodstream infections (C-RBSIs).

C-RBSI is a common complication, particularly among catheters placed in emergency departments, and is associated with poor outcomes, including in-hospital mortality (1–3). In one study including patients who received a central line catheter insertion in the emergency department, 3.7% experienced a C-RBSI during their hospital stay (2). Although the incidence of C-RBSI associated with central venous catheters (CVC) seems to be decreasing, they remain the primary source of C-RBSIs (4, 5). Meanwhile, there is an increasing incidence of C-RBSI involving peripherally inserted catheters (5). These nosocomial and healthcare-related infections are estimated to have an impact on hospital costs of approximately 18,000 euros per episode (6).

Traditionally, a definitive diagnosis of C-RBSI has depended on removing the catheter and culturing its tip (CC) using quantitative or semi-quantitative methods. However, a study indicated that CC results have limited clinical value, showing low sensitivity for diagnosing C-RBSI, minimal impact on patient mortality, and are not cost-effective (7). Moreover, another study including patients with positive CC but without bacteremia showed that antimicrobial therapy did not reduce the risk of subsequent infection or mortality in critically ill patients. This suggests that a positive CC result alone may not be a sufficient justification for initiating antibiotic treatment (8).

To prevent the unnecessary disposal of catheters and minimize complications from inserting new devices or lacking necessary ones, various conservative diagnostic procedures that do not require catheter removal have been developed (4, 9). The standard conservative procedure for the diagnosis of C-RBSI is the differential time to positivity (DTTP) technique. A difference in the time to positivity of 120 minutes between blood obtained from the catheter and blood obtained from the peripheral vein has been defined as a cutoff to microbiologically confirm C-RBSI (6, 10, 11). Even though DTTP may help clinicians anticipate the diagnosis of C-RBSI, there are still some concerns regarding this approach, as it has shown variability in effectiveness when dealing with C-RBSI caused by specific microorganisms, such as *Candida* spp. and *Staphylococcus aureus* (12–18). This might be explained by the early dissemination of cells in *C. albicans* and *S. aureus* biofilms, making the 120-minute threshold inaccurate for these microorganisms (19).

Moreover, a recent study showed poor sensitivity and limited specificity when applying this technique to confirm the clinical suspicion of C-RBSI in patients admitted to intensive care units (ICUs) (20). Consequently, it remains uncertain whether DTTP should be universally applied in all suspected cases of C-RBSI, irrespective of the pathogen involved.

The aim of this study was to evaluate the effectiveness of DTTP in accurately diagnosing C-RBSI within our institution.

## MATERIALS AND METHODS

This retrospective study was carried out in a 1,550-bed tertiary teaching hospital in Madrid, Spain, from October 2022 to July 2023 and included all C-RBSI episodes in which both DTTP BC and catheter culture (CC) were obtained. Only one episode per patient was selected, primarily including long-term catheters. We analyzed microbiological and clinical data, which were retrieved from the hospital’s electronic database.

### Catheter culture

For the CC, we routinely used the semi-quantitative Maki’s technique. Briefly, it consists of rolling the catheter tip forth and across a blood agar plate. Colonization is defined when ≥15 cfu/plate is recovered after 24–48 hours of incubation at 37°C under aerobic conditions. However, when endoluminal colonization occurs, it can be misdiagnosed by this technique. Therefore, when the CC was negative but there was still a high clinical suspicion of C-RBSI, we sonicated the catheter (after cutting the catheter tip into 2–5 mm fragments) into 10 mL of enrichment broth, followed by culture using a cutoff of 100 cfu/mL (21). In our laboratory, sonication is performed only when CC is negative but clinical suspicion of C-RBSI remains, or for sliced silicone catheters from neonates. The hospital’s electronic database showed no instances in which catheter sonication converted a negative CC result into a positive one.

### Pathogen identification

For pathogen identification, matrix-assisted laser desorption ionization–time of flight mass spectrometry (MALDI-TOF MS, Bruker Daltonics) was used directly from grown colonies, as previously described (22).

### Definitions

- C-RBSI by DTTP: A positive peripheral BC and BC collected through catheter lumen(s) for the same microorganism, with a difference between catheter-collected and peripheral BC growth of ≥2 hours, with or without positive catheter culture (DTTP+ CC+/DTTP+ CC−).
- Non-C-RBSI by DTTP: A positive peripheral BC and BC collected through catheter lumen(s) for the same microorganism, with a difference between catheter-collected and peripheral BC growth of <2 hours, with or without positive catheter culture (DTTP− CC+/DTTP− CC−).
- C-RBSI with CC (gold standard): A positive peripheral BC and catheter tip culture (>15 cfu) (in ports, also reservoir port culture) with isolation of the same microorganism (DTTP+ CC+/DTTP− CC+/peripheral BC+ CC+).
- Peripheral blood culture with CC: A peripheral BC with CC but without blood withdrawn from the catheter lumen (not included in the study).

### Statistical analysis

Qualitative clinical variables were expressed as numbers (percentage) and compared using the chi square test. Quantitative clinical variables were expressed as mean (standard deviation) and compared using the Student’s *t*-test. Significance was set at *P* < 0.05.

Comparisons between groups were assessed using the Kruskal–Wallis test, and statistical significance was set at *P* < 0.05.

We analyzed validity values of the DTTP technique to diagnose C-RBSI compared to the gold standard.

All tests were performed using SPSS Statistics for Windows, v.21.0 (IBM Corp, Armonk, New York, USA).

## RESULTS

**Fig 1:**
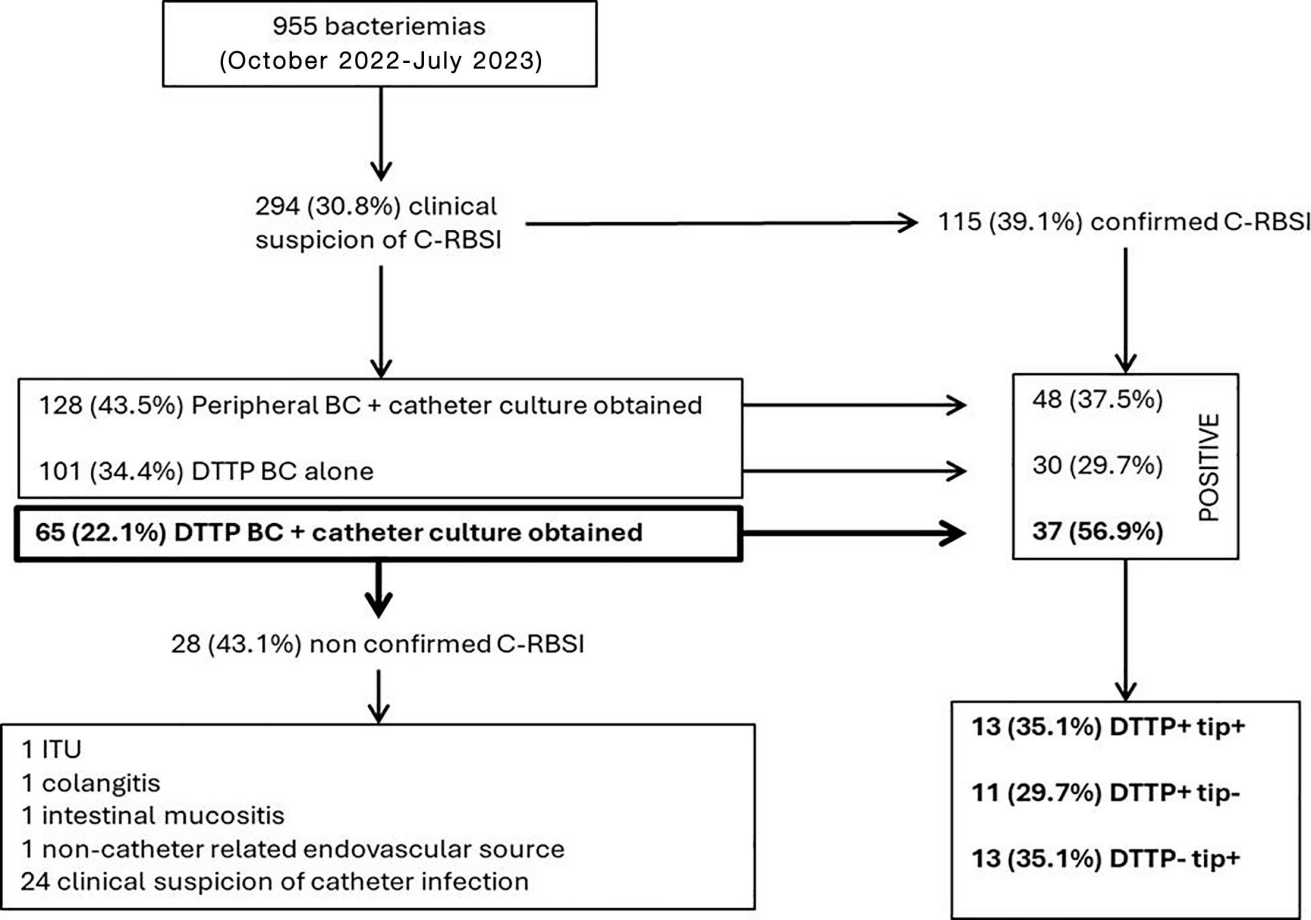
Algorithm of the study.

During the 9-month study period, we collected a total of 955 episodes of bacteremia, of which 294 (30.8%) were clinically suspected to be C-RBSI. Microbiological confirmation was achieved in only 115 cases (115/294; 39.1%) using any of the following methods: DTTP BC alone (30 episodes), DTTP BC with CC (37 episodes), or peripheral BC with CC (48 episodes). Out of the 294 episodes in which C-RBSI was clinically suspected, both DTTP BC and CC were obtained in only 65 (22.1%). Among the confirmed C-RBSI cases, both DTTP BC and CC samples were obtained in just 37 episodes (37/115; 32.7%), which were therefore included in the study (Fig. 1).

Among the 37 episodes, only 13 (35.1%) were positive for both DTTP BC and CC (with a difference between catheter and peripheral BC growth of ≥2 hours), while DTTP BC alone or DTTP BC with CC (with a difference between catheter lumen and peripheral BC growth of <2 hours) were positive in 11 (29.7%) and 13 (35.1%) episodes, respectively (Fig. 1).

Based on these data, the validity values of DTTP to detect C-RBSI were as follows: sensitivity, 50.0%; specificity, 71.8%; positive predictive value, 54.2%; and negative predictive value, 68.3% (Table 1).

**TABLE 1:** Diagnostic performance of DTTP BC for C-RBSI compared to CC as the gold standard in the 65 cases where both DTTP BC and CC were obtained^*a*^

Test result	C-RBSI confirmed by CC (gold standard)	C-RBSI not confirmed by CC (gold standard negative)	Total
DTTP BC positive	13 (TP)	11 (FP)	24
DTTP BC negative	13 (FN)	28 (TN)	41
Total	26	39	65

^*a*^ C-RBSI, catheter-related bloodstream infection; CC, catheter culture; DTTP BC; differential time to positivity blood culture; TP, true positive; FP, false positive; TN, true negative; FN, false negative.

More than a quarter of the patients had hematologic malignancies (27.0%), followed by those with solid organ tumors (16.2%) and solid organ transplantations (10.8%). Most of the cultured catheters were central venous catheters (CVC) (40.5%) and peripherally inserted central venous catheters (PICC) (29.7%). All patients with positive DTTP BC but negative CC had received antibiotics for at least 48 hours before catheter withdrawal. There were no differences among groups regarding the duration of antibiotic treatment before sample collection (CC), with an overall median (IQR) of 4 (3, 4) days, distributed as follows: DTTP+ CC+ group, 4 (3, 4); DTTP+ CC− group, 4 (3, 4); and DTTP− CC+ group, 4 (3–5). The most important clinical characteristics of the patients are shown in Table 2.

**TABLE 2:** Clinical characteristics of the patients^*a*^

Characteristic	Patients, *N* = 37
Median (IQR) age (years)	52.6 (18.7–63.5)
Male sex, *N* (%)	23 (62.2)
Underlying disease, *N* (%)	
Hematologic tumor	10 (27.0)
Solid organ tumor	6 (16.2)
Solid organ transplant	4 (10.8)
Surgery	3 (8.1)
Kidney disease	3 (8.1)
Respiratory disease	3 (8.1)
Other	8 (21.6)
Type of catheter, *N* (%)	
CVC	15 (40.5)
PICC	11 (29.7)
Port-A-Cath	9 (24.3)
PVC	1 (2.7)
Arterial	1 (2.7)
Diagnosis of C-RBSI, *N* (%)	
DTTP+ catheter culture+	13 (35.1)
DTTP+ catheter culture −	11 (29.7)
DTTP− catheter culture+	13 (35.1)
Median (IQR) days between bacteremia and catheter culture	2 (1–3)
C-RBSI etiology (39 isolated microorganisms), *N* (%)	
CoNS	12 (32.4)
*Candida* spp.	8 (21.6)
*Enterobacteriaceae*	8 (21.6)
Non-fermentative Gram-negative bacilli	6 (15.4)
*Staphylococcus aureus*	2 (5.4)
*Enterococcus faecalis*	1 (2.7)
*Streptococcus viridans*	1 (2.7)
*Clostridium* spp.	1 (2.7)
i.v. antimicrobial therapy, *N* (%)	37 (100)
Median (IQR) DDDs	21 (14–24)
Duration of i.v. antimicrobial therapy prior to sample collection (CC), median (IQR) days	4 (3–4)
Catheter lock therapy, *N* (%)	9 (24.3)
ICU admission, *N* (%)	2 (5.4)

^*a*^ IQR, interquartile range; CVC, central venous catheter; PICC; peripherally inserted central catheter; PVC; peripheral venous catheter; C-RBSI, catheter-related bloodstream infection; DTTP, differential time to positivity; CoNS, coagulase-negative staphylococci; i.v., intravenous; DDDs, defined daily doses; CC, catheter culture; ICU, intensive care unit.

The etiology of the 37 C-RBSI episodes was distributed as follows: Gram-positive bacteria, 43.6%; Gram-negative bacteria, 33.3%; and yeasts, 20.5% (Table 3). There were no statistically significant differences in the distribution of the microorganisms among the three groups.

**TABLE 3:** Microorganisms causing C-RBSI episodes detected by DTTP and/or CC^*a*^

Microorganism	*N* (%)			
	DTTP+ CC+	DTTP+ CC−	DTTP− CC+	Total
Gram-positive	6 (46.2)	6 (50.0)	5 (35.7)	17 (43.6)
*Staphylococcus epidermidis*	3 (23.1)	1 (8.3)	4 (28.6)	8 (20.5)
CoNS	2 (15.4)	2 (16.7)	0 (0.0)	4 (10.3)
* Staphylococcus aureus*	1 (7.7)	1 (8.3)	0 (0.0)	2 (5.1)
*Enterococcus faecalis*	0 (0.0)	0 (0.0)	1 (7.1)	1 (2.6)
*Streptococcus viridans*	0 (0.0)	1 (8.3)	0 (0.0)	1 (2.6)
*Clostridium sporogenes*	0 (0.0)	1 (8.3)	0 (0.0)	1 (2.6)
Gram-negative	4 (30.8)	4 (33.3)	6 (42.9)	14 (33.3)
*Klebsiella pneumoniae*	1 (7.7)	2 (16.7)	0 (0.0)	3 (7.7)
*Pseudomonas aeruginosa*	0 (0.0)	1 (8.3)	2 (14.3)	3 (7.7)
*Enterobacter cloacae*	0 (0.0)	0 (0.0)	2 (14.3)	2 (5.1)
*Escherichia coli*	0 (0.0)	0 (0.0)	1 (7.1)	1 (2.6)
*Klebsiella oxytoca*	1 (7.7)	0 (0.0)	0 (0.0)	1 (2.6)
*Serratia marcescens*	1 (7.7)	0 (0.0)	0 (0.0)	1 (2.6)
* Stenotrophomonas maltophilia*	1 (7.7)	0 (0.0)	1 (7.1)	2 (5.1)
*Pantoea septica*	0 (0.0)	1 (8.3)	0 (0.0)	1 (2.6)
Yeasts	3 (23.1)	2 (16.7)	3 (21.4)	8 (20.5)
*Candida albicans*	1 (7.7)	0 (0.0)	2 (14.3)	3 (7.7)
*Candida lusitaniae*	1 (7.7)	0 (0.0)	1 (7.1)	2 (5.1)
*Candida dubliniensis*	0 (0.0)	2 (16.7)	0 (0.0)	1 (2.6)
*Candida parapsilosis*	1 (7.7)	0 (0.0)	0 (0.0)	2 (5.1)
Overall	13 (33.3)	12 (30.8)	14 (35.9)	39 (100)

^*a*^ C-RBSI, catheter-related bloodstream infection; CoNS; coagulase-negative staphylococci; DTTP, differential time to positivity; CC, catheter culture. There were two polymicrobial infections: 1 episode of *S. maltophilia* + *Enterobacter cloacae* infection and 1 episode of *Pantoea septica* + *Candida dubliniensis* infection.

## DISCUSSION

This study examined the performance of the DTTP technique for diagnosing C-RBSI compared to the reference method of CC. A key finding is that the DTTP method failed to identify C-RBSI in over a third of cases where both DTTP and CC were obtained, meaning that without catheter removal and culture, microbiological confirmation would have been missed.

Our results demonstrated that the DTTP method showed a sensitivity of 50% and specificity of 71.8% to predict C-RBSI, raising concerns about the risk of false negatives, thereby leaving the active source of infection untreated. There were 11 cases (29.7%) in which CC was negative, potentially due to prior antibiotic use before catheter removal, which may have impacted bacterial detection (Table 1).

DTTP relies on the premise that blood drawn through the catheter will have a higher bacterial load compared to peripheral blood, but various factors (timing of blood collection, volume, and microbial species) can influence its accuracy (6, 23–28).

Previous studies evaluating the DTTP method primarily focused on oncologic or critical care patients and have reported mixed results. For example, Blot et al. demonstrated high sensitivity and specificity, both above 90%, for long-term catheters using a DTTP threshold of ≥120 minutes in a cohort of oncologic patients admitted to an intensive care unit (29).

Another study conducted in oncologic patients, including 108 episodes of C-RBSI, showed also a good performance of DTTP for diagnosing C-RBSI. A DTTP of ≥120 minutes was associated with a sensitivity and specificity of 81% and 92% for short-term catheters and 93% and 75% for long-term catheters, respectively. However, specificity significantly decreased in patients who had received antibiotics (26). Similarly, a study by Bouza et al., comparing three different methods for the diagnosis of C-RBSI without catheter withdrawal in ICU patients with short-term catheter use, found sensitivity and specificity above 90% for DTTP (27).

On the other hand, other research has shown more variable outcomes depending on catheter type, prior antibiotic use, and the organisms involved. For instance, a study by Rijnders et al., conducted in an ICU setting, found no significant differences in DTTP between patients with C-RBSI and those with non-catheter-related bacteremia (30). Similarly, Kaasch et al. aimed to assess the effectiveness of DTTP in diagnosing C-RBSI caused by *Staphylococcus aureus*. Their study reported a low sensitivity of 37% and a specificity of 77%, suggesting that clinicians should not rely on DTTP alone for diagnosis (31). Orihuela et al. found that DTTP was effective for diagnosing C-RBSI only in cases involving AmpC-producing *Enterobacteriaceae* and *Pseudomonas aeruginosa* (sensitivity of 76% and 87%, respectively). However, its performance was notably poorer in diagnosing infections caused by common Gram-positive organisms and non-AmpC-producing enteric bacilli (9). Likewise, in a recent five-year, single-center retrospective study in an ICU, Bisantti et al. reported a sensitivity of 40.5% and a specificity of 73.7% for DTTP (20).

These last studies align with the findings of our work, further underscoring the limitations of DTTP in reliably diagnosing C-RBSI across various clinical settings and bacterial pathogens.

One of the strengths of our study is that, unlike the majority of the studies mentioned above, less than half of the included patients suffered from hematological or solid organ malignancies, and only two were patients admitted to the ICU.

Most of the microbiological isolates identified were either Gram-positive or Gram-negative bacteria, and DTTP demonstrated similar performance in both groups. In eight episodes, yeasts were isolated; however, DTTP failed to classify three of these cases (37.5%) as catheter-related bloodstream infections (C-RBSI). This result is consistent with previous studies suggesting that the standard 2-hour threshold may not be sufficient for accurately detecting yeast-related infections (13, 14, 17).

The study has several limitations. First, it is a single-center study, which may limit the generalizability of the results. However, procedures were conducted according to standardized and well-defined protocols at our institution, which should enhance the reproducibility of our findings. Second, although the number of episodes included is relatively small, it reflects real-world clinical practice and highlights that, even when C-RBSI is suspected, the combination of CC and DTTP blood culture is not commonly performed; in our study, this approach was implemented in only 22.1% of suspected C-RBSI episodes. Third, the volume of blood withdrawn in each culture bottle is known to influence results. While we assumed that the amount collected met standard guidelines (32), this was not specifically controlled in our study. Fourth, the use of antibiotics prior to catheter withdrawal could have affected the CC, leading to false-negative results. Additionally, our study primarily included long-term catheters, limiting the applicability of the findings to short-term catheter cases.

### Conclusions

This study highlights that DTTP is a useful conservative technique for diagnosing C-RBSI, particularly when catheter removal is not an option. However, as shown in previous studies, its accuracy can be influenced by factors such as the timing and volume of blood collection, as well as the type of microorganism involved. Therefore, DTTP results should be interpreted with caution and not relied upon exclusively to rule out C-RBSI. Catheter culture (CC), prior to starting antimicrobial therapy, remains the most reliable method for diagnosing and confirming C-RBSI.

## Data Availability

No primary research results, software, or code have been included and no new data were generated or analyzed as part of this review.

## AFTER EPUB

*[This article was published on 8 April 2025 with errors in the title. The title was corrected in the current version, posted on 17 April 2025.]*
